# Effect of Rearing Conditions on Growth, Fatty Acid Profile and Antioxidant Activity of Atlantic Salmon (*Salmo salar*)

**DOI:** 10.3390/ani16081139

**Published:** 2026-04-09

**Authors:** Md Zakir Hossain, Manpreet Kaur, Rachel M. Cole, Kevin J. Fisher, Sheryl Barringer

**Affiliations:** 1 Department of Food Science and Technology, The Ohio State University, Columbus, OH 43210, USA; hossain.154@buckeyemail.osu.edu (M.Z.H.); kaur.333@buckeyemail.osu.edu (M.K.); cole.311@osu.edu (R.M.C.); 2 School of Environment and Natural Resources, The Ohio State University, Columbus, OH 43210, USA; fisher.645@buckeyemail.osu.edu

**Keywords:** temperature, growth, lipid content, PUFAs, oxidative stress

## Abstract

Atlantic salmon (*Salmo salar*) is an important dietary source of health-promoting long-chain polyunsaturated fatty acids (PUFAs). As rearing conditions significantly influence fillet quality, this study evaluated the effects of warm and cool rearing temperature and photoperiod regimes on salmon growth, lipid profiles, and antioxidant capacity. Atlantic salmon (210 days old) were reared for 92 days under low temperature (14 °C, 12 h light) or high temperature (21 °C, 24 h light) conditions to simulate relevant seasonal conditions, winter and summer respectively. At day 302, conditions were reversed to create low-to-high (L→H) and high-to-low (H→L) treatments, continuing until day 362. Growth parameters, muscle lipid content, fatty acid profile, and antioxidant activity were measured at 302 and 362 days. Lipid content and fatty acid profile were also measured based on fillet location and fish sex. High rearing temperatures accelerated weight gain and increased total and neutral lipid contents, but elevated saturated fatty acids (SFA) and decreased PUFAs in structural polar lipids. High temperatures also significantly increased antioxidant activity, indicating elevated oxidative stress. Conversely, low temperatures suppressed growth but preserved essential PUFAs and maintained oxidative stability. Following the temperature shift, the H→L group had enriched polar lipids with PUFAs and maintained oxidative stability. On the other hand, L→H group showed lower PUFAs accumulation in polar lipid and enhanced oxidative stress. Total lipid content was higher in the head region, followed by the middle and tail sections of the fillet. However, fatty acid composition remained largely uniform across all three sections of the fillet. There were no significant differences in total lipid content between fish sexes. In conclusion, production efficiency and nutritional quality can be optimized by initially rearing salmon at high temperatures to promote rapid growth, followed by low temperature finishing phase to increase essential PUFA content and maintain oxidative stability.

## Simple Summary

Rearing conditions can affect Atlantic salmon growth, lipid quality, and oxidative stress levels, so adjusting rearing temperature and light is essential for optimal production. In this study, salmon were initially raised in either winter (14 °C, 12 h light) or summer (21 °C, 24 h light) conditions. Later, fish were switched to the opposite conditions to optimize production. The results highlighted that fish reared at high temperature (summer) grew faster and stored more lipids, but it also increased saturated fat levels and triggered higher oxidative stress. Conversely, cold water (winter) stimulated the fish to accumulate healthier polyunsaturated fatty acids (PUFAs). Overall, it was found to be effective to grow fish initially at high temperatures to achieve fast growth and then switch to low temperatures to increase essential PUFA content and maintain lowered oxidative stress.

## 1. Introduction

Atlantic salmon (*Salmo salar*), a representative of the family Salmonidae, is a rich source of long-chain omega-3 PUFAs, especially eicosapentaenoic acid (EPA) and docosahexaenoic acid (DHA) [1,2]. Higher dietary intake of these n-3 PUFAs has been linked to a reduced risk of chronic conditions such as cardiovascular disease and cognitive decline [3,4]. Maintaining the content of n-3 PUFAs of salmon is therefore not only a fish quality objective but has also broader public health importance. According to a report by the Food and Agriculture Organization of the United Nations [5], in the first quarter of 2025, worldwide salmon production was approximately 1,392,400 tonnes, representing an increase of 13.6% compared to the same period in 2024. In the first half of 2025, the United States imported 252,280 tonnes of salmon valued at 3.06 billion dollars. Per capita consumption of salmon in the United States has nearly doubled since 2010 to around 2 kg [5].

As the aquaculture industry scales to meet this demand, optimizing rearing protocols to accelerate growth without compromising fillet quality has become an operational challenge. Temperature is considered one of the major abiotic factors that affect fish growth [6,7,8], metabolism [7,9,10] and lipid composition [11,12,13,14]. During winter-like conditions, the optimal rearing temperature for juvenile and sub-adult salmon growth is typically around 14 °C [8,15], whereas in summer, temperatures can exceed 20 °C. These thermal variations are inherently coupled with drastic shifts in photoperiod. Winter environments are naturally characterized by restricted daylight (12 h of light), whereas summer conditions frequently expose fish to continuous, 24 h daylight [16,17]. Therefore, understanding how these coupled seasonal shifts in temperature and photoperiod interact to impact the overall growth performance, oxidative stress, and final fillet quality is critical for developing sustainable production strategies as well as to ensure fillet nutritional quality.

While elevated temperatures generally accelerate growth by enhancing metabolic activity, the recent literature suggests they may induce physiological trade-offs. Studies indicate that rearing salmon above optimal thermal thresholds can trigger oxidative stress, leading to the depletion of intracellular antioxidants like Vitamin C and E, and the accumulation of reactive oxygen species (ROS) [18,19]. Furthermore, homeoviscous adaptation theory suggests that fish reared in low temperatures can enhance membrane fluidity by increasing unsaturated fatty acids concentration [20]. Conversely, maintaining fish constantly at high temperatures can dilute these essential PUFAs, diminishing the nutritional profile that defines the commercial value of the fillet [20].

Intensive rearing practices combining high temperatures and continuous extended photoperiods offer distinct commercial advantages, primarily by elevating growth rate and accelerating weight gain [21]. However, these accelerated conditions often induce severe physiological trade-offs, often by elevating the metabolic rate and triggering severe oxidative stress [21]. Rapid mitochondrial respiration due to a high metabolic rate can increase ROS generation [6]. This directly causes the peroxidation of highly vulnerable PUFAs, ultimately yielding off-flavours and fillet degradation [22]. Apart from environmental conditions, the inherent factors of the fish, such as sex [23] and location within the fillet [24] used for analysis can also affect the lipid content and composition of fillets.

Although the effects of temperature and photoperiod on fish growth are well documented, critical knowledge gaps remain regarding temperature switches. It is currently unclear whether a reversal can be achieved, specifically whether salmon reared at high temperatures can recover their optimal PUFA profiles and quench oxidative stress when switched to a lower temperature. Therefore, the present study comprehensively evaluates the impacts of static temperature (low (14 °C) and high (21 °C)) and sequentially shifted (low-to-high, high-to-low) temperature and photoperiod regimes. Based on the established biological boundaries of this species, we designed our study to capture both ends of this critical spectrum: we selected 14 °C to represent optimal growing condition, and 21 °C to serve as a severe high-temperature condition pushing the fish to their upper thermal limits. By examining growth performance, spatial lipid allocation, fish sex, and endogenous antioxidant capacity, this study sought to identify a rearing strategy that balances optimal growth with superior nutritional fillet quality.

## 2. Materials and Methods

### 2.1. Experimental Design and Rearing Conditions

Before the study began, all procedures were approved by the Ohio State University Institutional Animal Care and Use Committee (2008A0220-R5, 2008A021-R5). The approval date was 14 April 2024. Mixed-sex Atlantic salmon (*Salmo salar*) embryos were procured from the U.S Department of Agriculture’s Agricultural Research Service facility (Leetown, WV, USA). The embryos were hatched at the Aquaculture Laboratory at the School of Environment and Natural Resources, The Ohio State University, in California trays (Mari Source, Burlington, WA, USA) to replicate river-like water flow conditions. After the yolk sac was absorbed, the juveniles were transferred to recirculating rearing tanks to initiate dry diet feeding (Advance 0.3–0.5 mm) (Alltech Coppens, Helmond, The Netherlands). The fish were reared for 210 days in recirculating tanks before the experiment began. After 210 days, fish were transferred to two 400 L round-bottom tanks (one single tank designated for the low-temperature group utilizing a recirculating aquaculture system, and one single tank designated for the high-temperature group utilizing a flow-through system). Due to facility space and infrastructure limitations at the time of the experiment, the experimental design was logistically restricted to utilizing a single tank for each treatment. The RAS setup was connected to multiple tanks through a mechanical filtration and sump pump system. The system allowed switching between chilled recirculation and flow-through fresh water via valves, enabling differential temperature control in parallel tanks without transferring fish. Two distinct rearing conditions were maintained to simulate variable aquaculture environments: low temperature (14 °C) with 12 h light–12 h dark cycle and high temperature (21 °C) with continuous light (24 h). To mimic natural seasonal conditions, high-temperature treatments were paired with 24 h photoperiods (simulating summer), while low-temperature treatments were paired with 12 h photoperiods (simulating winter). Combining temperature and photoperiod allowed simulation of seasonal conditions. Photoperiod conditions were maintained using artificial lighting. The tank was illuminated by a single 32-watt spiral fluorescent bulb (Agri Sales Inc., Ceresco, NE, USA) (2100 lumens, 5000 K daylight color temperature) suspended 1 m above the water surface. To ensure controlled light exposure, the lighting unit was fitted with an opaque cover that directed the illumination strictly downwards into the tank area.

During the experimental period, water quality parameters were continuously monitored daily to ensure a stable and optimal environmental baseline for the aquaculture system. The dissolved oxygen levels were maintained at 88.80 ± 6.63% of oxygen saturation level. Increase in water temperature showed inverse relationship with dissolved oxygen saturation level (*r* = −0.78). The pH level was maintained at 7.37 ± 0.16. Ammonium (NH_4_^+^) concentrations were 0.12 ± 0.04 mg/L. Unionized ammonia (NH_3_) and free chlorine were 0 mg/L and 0 ppm, respectively, throughout the study.

The mean weight of juvenile fish selected for the experiment was between 65 and 70 g. These fish were reared for 152 days under one of the two rearing conditions with a stocking density of 39 fish/tank on day 210, and 36 fish/tank on day 302. Fish were fed extruded and floating commercial feed (2–3 mm) (Skretting, Tooele, UT, USA) composed of 48% crude protein and 18% crude lipid. To eliminate feed restriction as a confounding variable, the fish were fed to apparent satiation (ad libitum) twice daily at 08:30 and 17:00 h using an automatic feeder. Any uneaten feed was subsequently collected and weighed, and this value was subtracted from the total feed dispensed to accurately quantify actual feed intake for calculating feed conversion ratio. After 302 total days of rearing, fish reared in low temperatures were acclimated to high temperatures, and those in high temperatures were acclimated to low temperatures. To mimic natural seasonal transitions, photoperiods were adjusted simultaneously with the temperature shifts. As these two environmental variables were altered concurrently to represent a single seasonal shift, they were analyzed statistically as a single combined factor rather than as independent variables.

### 2.2. Harvest and Sample Preparation

Fish were harvested at two sampling points (302 and 362 days) to collect fish for lipid analysis, maturity assessment, and growth measurement. Fish survival rate was monitored daily throughout the experimental period by visually inspecting the tanks and recording any mortality to calculate the final survival rate, which was 100%. At each sampling point, three fish were harvested from each rearing condition, and the fish were euthanized humanely by a blow to the head and immediately bled via dorsal artery severance. Weight, length, and gonad weight were measured for all the sampled fish. The fish were cut into two fillets; the skin and fillets were immediately stored at −80 °C for lipid analysis. Feed conversion rate (FCR), specific growth rate (SGR), gonadosomatic index (GSI) and condition factor (CF) were used to evaluate fish growth throughout the experiment.

SGR(%/day) = ln (Final body weight) − ln (Initial body weight)/Days of feeding × 100(1)

FCR = Feed given (g)/Weight gain of fish (g)(2)

Condition Factor (CF) = Weight (g)/Length^3^ (cm^3^) × 100 (3)

Gonadosomatic Index (GSI) = Gonad weight (g)/Total weight of fish × 100(4)

### 2.3. Lipid Extraction and Methylation

#### 2.3.1. Total Lipid Extraction

Total lipids were extracted from fish fillets, methylated, and quantified by gas chromatography with flame ionization detection (GC-FID) [25,26]. Fish muscle in the amount of 1.5 g was combined with 20 mL of a chloroform–methanol (Chem Products, Portland, OR, USA) mixture (2:1, *v*/*v*) and homogenized for 1–2 min using Omni GLH-115 (Omni International, Kennesaw, GA, USA). The mixture was filtered through Whatman No. 1 filter paper (11 µm) using vacuum filtration. To facilitate phase separation, 4 mL of saturated MgCl_2_·6H_2_O solution was added to the mixture. The mixture was vortexed for 1 min and then stored in specialized separation glassware for phase separation. The lipid layer was formed at the bottom and collected in glass tubes. These tubes were evaporated under nitrogen with mild heating and total lipid content was recorded.

#### 2.3.2. Determination of Neutral Lipid (NL) and Polar Lipid (PL) Content

A 20 mL syringe equipped with a Sep-Pak silica cartridge (Waters, Milford, MA, USA) filter tube was utilized to separate total lipids into polar (PL) and neutral (NL) lipid fractions. For NL extraction, 20 mL of chloroform was poured into the cartridge. Samples were introduced at the bottom of the syringe using a Pasteur pipette, followed by chloroform to collect the NL fraction. After chloroform completely passed through the syringe, the collection tube was replaced and 20 mL of methanol was used to extract the PL fraction. Extracted lipids were evaporated under nitrogen gas with heating.

#### 2.3.3. Lipid Methylation for GC Analysis

The NL fraction was processed initially, followed by the PL fraction. Chloroform was removed from the NL sample under nitrogen. Sodium hydroxide solution in methanol in the amount of 1.5 mL was added, and the mixture was incubated in an oven at 80 °C for 1 h. Samples were cooled at room temperature for 5 min. After 30 min of NL processing, PL samples were prepared in the same way. For both NL and PL, 2 mL of boron trifluoride (BF_3_) in methanol was added, and samples were incubated at 80 °C for 30 min. After cooling, 1 mL of hexane and 1 mL of water were added to each sample, and the mixture was vortexed for 1 min. The upper hexane phase was collected into tubes containing anhydrous sodium sulfate (to remove residual water and impurities). An additional 1 mL of hexane was added, and the phase was collected again into tubes containing sodium sulfate. The final hexane extracts were transferred into amber glass vials for GC analysis.

Analysis of fatty acid methyl esters was performed by gas chromatography with a flame ionization detector using a 30 m Omegawax TM 320 fused silica capillary column (Supelco, Bellefonte, PA, USA) and helium as the carrier gas. The oven temperature started at 175 °C, increased at 3 °C/min until reaching 220 °C, and then decreased at 5 °C/min until reaching 210 °C. Retention times of the samples were compared with standards for fatty acid methyl esters (Matreya, LLC, Pleasant Gap, PA, USA and Nu-Check Prep Inc., Elysian, MN, USA). Fatty acids are reported as a percentage of the total identified fatty acid as previously reported [27,28,29].

### 2.4. Antioxidant Activity Analysis

#### 2.4.1. ABTS Radical Scavenging Assay

Fish fillet sample (1 g) was homogenized with 7 mL of n-hexane/ethanol (5:2) and centrifuged at 4000 rpm for 5 min. Centrifugation resulted in two phases (hexane on top, ethanol on bottom); the lower ethanol phase was collected and used for all subsequent antioxidant assays. The ABTS radical scavenging activity (RSA) was determined by the following method, with slight modifications [30]. The ABTS radical cation (ABTS·^+^) was generated by mixing 7 mM ABTS with 2.45 mM potassium persulfate and incubating at room temperature for 16 h. For the analysis, 2 mL of the diluted ABTS·^+^ solution was mixed with 100 μL of the sample, incubated in the dark for 30 min, and the absorbance was measured at 734 nm using a spectrophotometer.

RSA (%) = (A_control_ − A_sample_)/A_control_ × 100 (5)

where A_control_ and A_sample_ are the absorbance values of the control and test samples, respectively.

#### 2.4.2. DPPH Radical Scavenging Assay

The DPPH radical scavenging activity (RSA) of fish muscle was assessed by mixing 100 μL of the sample with 2 mL of 0.1 mM DPPH solution in methanol [18]. The mixture was incubated in the dark at room temperature for 30 min, and the absorbance was measured at 517 nm.

#### 2.4.3. Ferric Reducing Antioxidant Power (FRAP)

The FRAP assay was conducted following [18,31] methods with minor modifications. Sample extract in the amount of 0.5 mL was mixed with 0.5 mL of 1% potassium ferricyanide and 0.5 mL of phosphate buffer. After incubation at 50 °C for 20 min, 0.5 mL of 10% (*w*/*v*) trichloroacetic acid was added. Samples were incubated at room temperature for 30 min. The supernatant (1 mL) was mixed with 0.2 mL of 0.1% ferric chloride. The solution was incubated for 10 min, and the absorbance was measured at 700 nm.

### 2.5. Statistical Analysis

The main effects of the primary temperature–photoperiod treatments on growth performance, lipid content, fatty acid composition, and antioxidant capacity were analyzed using one-way analysis of variance (ANOVA) with a sample size of *n* = 3. Additionally, two-way ANOVA was used to separate the effects of sex and spatial distribution of fatty acid profile from temperature effect. When significant differences were identified, Tukey’s Honestly Significant Difference (HSD) post hoc test was applied for multiple mean comparisons. A *p*-value of less than 0.05 was considered significant in all analyses. It is important to note that, due to facility space constraints, the experimental design is limited by one tank per treatment. The sampled fish represent observational units within their respective tanks rather than true independent experimental replicates, which can cause pseudoreplication [32]. Therefore, the reported *p*-values reflect within-tank variance rather than true between-treatment variance. JMP Student Edition 18 (JMP Statistical Discovery LLC, Cary, NC, USA) and Microsoft Excel were used for the statistical analysis. All data were expressed as mean ± SD.

## 3. Results and Discussion

### 3.1. Growth Performance

In this study, rearing temperature and photoperiod were altered simultaneously to simulate seasonal changes (winter (14 °C, 12 h light) and summer (21 °C, 24 h light) conditions). As these environmental variables were coupled, their independent effects cannot be statistically isolated. Recent research demonstrates that Atlantic salmon (*Salmo salar*) growth is significantly more affected by the limiting effects of water temperature than by photoperiod manipulation [33]. Therefore, the growth differences observed between the seasonal regimes in this study are primarily attributed to thermal variation.

The result of this study showed that lower rearing temperature significantly reduced growth in salmon. At 302 days, fish reared in low temperature showed lower body weight (184 g) and length (27 cm) than those reared in high temperature: body weight (328 g) and length (30 cm) (Table 1). After 302 days, the fish were switched to the other rearing temperature (low-to-high, high-to-low). At 362 days, growth was consistent with the temperature effects observed earlier, with fish transferred from low to high temperature (L→H) gaining an additional 156 g, whereas fish transferred from high to low temperature (H→L) gained only 92 g. The accelerated growth at high temperatures is due to an increase in metabolic rate and feed utilization [8,34]. Atlantic salmon previously showed enhanced growth at 15 °C compared with 8 °C, demonstrating the positive effect of elevated temperature on growth performance [11]. Similar temperature effects have been reported in a related species, Chinook salmon, which showed better growth performance at 16 and 20 °C than at 8 or 12 °C [35]. Low rearing temperatures prolong gut transit time and lower feed utilization which subsequently hinders growth [8,36].

Similarly, fish reared at low temperature showed lower specific growth rate (SGR; 1.20% day^−1^) than those at high temperature (1.75% day^−1^) (Table 1). Following the temperature shift, L→H fish slightly increased SGR (1.28% day^−1^), demonstrating growth compensation in response to higher temperature, while H→L fish had reduced SGR (0.41% day^−1^), indicating strong growth suppression at low temperatures. These results highlight the strong dependence of growth rate on thermal conditions. Consistent with these findings, another study reported substantially higher SGR in salmon reared at 15 °C compared with 8 °C, underscoring the positive effect of high temperature on growth performance [11].

In addition, feed conversion ratio (FCR) was higher at low temperature than at high temperature, indicating that fish reared at low temperatures required more feed to achieve equivalent weight gain (Table 1). Similarly, when the temperature was switched (L→H), FCR decreased and (H→L) FCR increased, suggesting that higher temperatures promote more efficient feed utilization. The observation of a higher FCR at low temperatures is attributed to reduced thermodynamic energy, which decreases the activity of digestive enzymes and lowers overall nutrient assimilation efficiency. Conversely, the low value (improved) FCR at high temperatures is driven by accelerated digestive enzyme activity [37]. In the literature, the temperature effect on FCR is complicated. One study found that Chinook salmon had high FCR at 20 °C, low at 16 and 12 °C and high at 8 °C [35]. Another study found an interaction between temperature and body weight, with better feed utilization at 13 °C for Atlantic salmon weighing 70–150 g and at 11 °C for Atlantic salmon weighing 170–300 g [8].

Condition factor (CF) is commonly used as an indicator of body form. CF values of 1 represent average condition under isometric growth, with lower values indicating a thinner body form, and higher values reflecting greater tissue and energy reserves [38]. In this study, low rearing temperatures produced lower CF, reflecting limited deposition of tissue and energy reserves (Table 1). The CF value was 0.9 in the low temperature group compared to 1.11 in the fish exposed to high temperature (Table 1). A similar pattern was observed following the temperature shifts, with CF increasing in fish transferred L→H and decreasing in fish transferred H→L. These results indicate that low temperatures reduce CF values, while high temperatures increase them. Consistent with these results, CF value was reported to increase with rising rearing temperature (8–20 °C) in Chinook salmon, reflecting enhanced body form at warmer temperatures [35].

Gonadosomatic index (GSI) data indicated that both male and female fish exhibited lower gonadal development at low rearing temperatures (Table 1). Transferring to L→H regimes increased GSI for female fish. Consistent with our findings, higher maturation rates have been reported at 12 °C compared with 8 °C [21]. This temperature-driven acceleration of gonadal development is mediated by the brain–pituitary–gonad (BPG) endocrine axis in fish. Higher temperatures accelerate the release of hormones (gonadotropins) that drive rapid gonadal growth [39]. From a physiological perspective, this early maturation has significant metabolic implications; it forces the fish to reallocate energetic resources and lipid reserves away from somatic muscle growth and toward reproductive tissues, which can ultimately compromise final fillet yield [40].

Overall, low temperature combined with a 12 h light period impaired the growth rate, feed conversion efficiency, condition factor, and gonadal development. These responses were consistent following switching of the temperature, highlighting temperature as a significant factor of growth, feed efficiency and gonadal development in this species.

### 3.2. Muscle Lipid Content

Low rearing temperatures produced lower total and neutral lipid content, with no significant difference in polar lipid content (Table 2). Total lipid content was almost half (2.7%) in fish reared at low temperatures compared to those reared at high temperatures (4.6%). As the fish gained weight at high temperatures, they also accumulated more lipids. This temperature effect was driven by the neutral lipid fraction, which was higher at high temperature (3.56%) than at low temperature (1.66%), indicating enhanced accumulation of storage lipids. After switching temperatures, H→L fish showed higher total lipid content (6.9%) than L→H (5.26%), suggesting that the initial high levels of lipids from growing at high temperatures remained the dominant effect even after switching to a lower temperature, and the fish that started at a lower temperature did not catch up when moved to a higher temperature. Again, the difference was in neutral lipids, with more neutral lipids in H→L fish (5.18%) than in L→H (4.05%). Previous studies have similarly demonstrated lower lipid content in Atlantic salmon juveniles at lower temperature (10 °C) compared to higher temperature (20 °C) [41].

In contrast, there was no significant difference in polar lipid content in any rearing condition, suggesting that structural lipids in muscle were conserved across rearing temperatures (Table 2). Rearing temperature has previously been shown to have limited influence on polar lipid quantity in Atlantic salmon muscle [12]. These results indicate that rearing temperature influences muscle total lipid content through changes in neutral lipid accumulation, while polar lipid content remains relatively stable, as also shown in the literature [12,20].

### 3.3. Muscle Fatty Acid Profile

The fatty acid composition was more affected by rearing temperature in the polar lipid fraction than in the neutral lipid, indicating a stronger temperature effect on polar (membrane) lipid composition than on neutral (storage) lipid composition (Table 2).

The polar lipid fatty acid composition showed a temperature-dependent pattern driven by the distinct biochemical regulation of individual fatty acids (Table 2). Fish reared at low temperatures exhibited higher PUFA and lower SFA compared with fish reared at high temperatures, resulting in a significantly higher PUFA/SFA ratio (2.03 vs. 1.73). Similarly, after temperature switching, fish shifted from high to low temperatures (H→L) increased their PUFA and reduced their SFA levels. However, this thermal-shifting strategy (H→L) presents a distinct growth and nutritional quality trade-off. While lowering the rearing temperature successfully provided a more favourable nutritional lipid profile, it did so at the expense of growth rate (Table 1). The sudden reduction in temperature suppressed metabolic and digestive enzymatic activity, resulting in a significantly lower growth rate and poorer feed efficiency (Table 1) compared to the continuous high-temperature regime. While thermal shifting to low temperature can incorporate more PUFAs in fish muscle, producers must carefully weigh this nutritional benefit against the losses in production efficiency.

This overall compositional change stems from the selective incorporation of specific fatty acids. At high temperature, the proportion of palmitic acid (C16:0) in the polar fraction significantly increased to 24%, compared to only 21% at low temperature, and stearic acid (C18:0) exhibited a similar upward trend (Table 2). One of the important functions of these saturated fatty acids is to maintain the integrity of the cell membrane across varying thermal conditions. At high temperatures, the kinetic energy of the lipid bilayer increases, risking hyper-fluidity and potentially damaging cellular membranes. To counteract this, fish incorporate higher proportions of straight-chain SFAs. As these SFAs lack double bonds, they pack tightly together, increasing the rigidity of the phospholipid bilayer to prevent cellular destabilization [13,42].

Conversely, colder temperatures cause cell membranes to stiffen. To maintain viability, colder temperatures favoured the incorporation of essential long-chain n-3 PUFAs. The multiple double bonds in PUFAs create bends in their hydrocarbon chains, which physically prevents the lipids from packing too tightly [42,43]. Specifically, docosahexaenoic acid (DHA, C22:6n-3) accumulated in higher proportions at low temperatures, accounting for 37% of the polar lipids, whereas it dropped to 30% in the group shifted from low to high temperatures (L→H) (Table 2). The n-3/n-6 ratio was also higher at low temperature, reflecting a greater content of essential n-3 PUFAs, e.g., eicosapentaenoic acid (EPA, C20:5n3) and docosahexaenoic acid (DHA, C22:6n3) which declined under high temperatures [20,44,45]. The increased accumulation of DHA at low temperatures is driven by an upregulation of endogenous elongation and desaturation pathways, which are naturally stimulated in cold environments to meet biophysical membrane demands [34]. These temperature-dependent shifts in polar lipid fatty acid composition are consistent with homeoviscous adaptation, where increased PUFA incorporation at low temperatures enhances membrane fluidity, while higher temperatures favour increased saturation to maintain membrane stability [46].

In contrast, in neutral lipids, there were no significant differences in SFA, monounsaturated fatty acids (MUFAs) and PUFA proportions in fish reared at any temperature. The major representative fatty acids—such as SFA-like palmitic acid (C16:0), MUFA-like oleic acid (C18:1n9) and PUFA-like EPA (C20:5n3) and DHA (C22:6n3) were maintained at similar percentages regardless of temperature (Table 2). This uniformity occurs because neutral lipids (triacylglycerols) function as metabolic storage reservoirs rather than functional structural components [1]. Because the salmon in this study were fed an identical diet, their bulk storage lipids mirrored the dietary profile, agreeing with previous studies showing that temperature has relatively minor effects on the fatty acid composition of storage lipids [7,12].

Overall, these findings indicate that rearing temperature influences both lipid quantity and lipid quality in salmon muscle. High temperatures increased neutral lipid deposition, whereas lower temperatures favoured modifications of membrane lipids, characterized by higher proportions of PUFAs in muscle.

### 3.4. Effect of Spatial Variation in Fatty Acid Profile

The internal distribution of lipids within the salmon fillet was heterogeneous along the anteroposterior axis (Table 3). Total lipid, neutral lipid, and polar lipid contents were highest in the muscle close to the head and lowest in the tail region, demonstrating a clear anteroposterior (head-to-tail) gradient in lipid deposition. Similar spatial patterns of lipid distribution along the fillet axis have been reported previously in salmon, with the head region acting as a primary lipid storage site and the tail region remaining comparatively lean due to involvement in muscle movement [24,47].

The fatty acid composition of the neutral and polar lipids was largely conserved across fillet regions, with most individual fatty acids and major fatty acid classes showing no significant spatial variation (Table 3). This agrees with previous studies demonstrating that spatial heterogeneity in salmon fillets is mainly attributable to differences in lipid quantity rather than changes in the fatty acid composition [48]. In this study, the n-3/n-6 ratio was higher in the tail region in both the polar and neutral lipid fractions, indicating a localized shift in essential fatty acid profiles. Furthermore, docosahexaenoic acid (DHA, C22:6n-3) was significantly higher in the neutral lipids of the tail region compared to the anterior sections. Previous study on Atlantic salmon also showed higher proportion of DHA in tail region, suggesting targeted deposition of DHA in active tail muscle [24]. The active tail muscle also contains a high density of mitochondrial cells to meet the energy demand of this active tissue. As DHA is a critical structural component of mitochondrial membranes, they were presented in higher concentration in tail than other sections of the fillet [24,42].

Overall, the findings demonstrate that fillet location strongly influences lipid quantity (head-to-tail) and has less effect on individual fatty acid composition. However, it has effect on deposition of essential n-3 polyunsaturated fatty acids in the active muscle regions.

### 3.5. Lipid Profile Based on Fish Sex

Total lipid, neutral and polar lipid content were not significantly different between male and female fish, although there was a trend to accumulate more total and neutral lipid in male fish (Table 4). Studies on Atlantic salmon juveniles showed slightly higher total lipid levels in the muscle tissues of males than females [23]. The analysis did not show significant differences in the broad classes of SFAs, MUFAs and PUFAs between the sexes.

Despite similarities in gross lipid classes, two specific fatty acids highlight a distinct biochemical difference between the sexes, driven by reproductive needs. Specifically, the concentrations of gondoic acid (C20:1n-9) and EPA (C20:5n-3) serve as indicators for sexual maturity. In the neutral lipid fraction, females exhibited a significantly higher concentration of gondoic acid compared to males. An increase in this particular monounsaturated fatty acid indicates an increase in female sexual maturity and preparation for ovary development [49]. In polar lipids, males exhibited a significantly higher EPA concentration, a key long-chain PUFA. Males specifically increase the concentration of this essential PUFA within their structural membranes to support sperm production [50]. Therefore, while overall lipid class totals remain similar, the sex-specific elevation of gondoic acid in females and EPA in males reflects their distinct physiological preparations for reproduction.

### 3.6. Antioxidant Activity of Fish Muscle

Salmon reared at different temperatures showed significant differences in muscle antioxidant capacity as measured by ABTS, DPPH, and FRAP assays (Table 5). Fish muscle is a complex matrix containing antioxidants that neutralize reactive oxygen species via distinct chemical pathways, primarily Single Electron Transfer (SET) and Hydrogen Atom Transfer (HAT). Relying on a single assay would systematically underestimate the muscle total antioxidant capacity. Therefore, we employed a complementary analysis: FRAP (SET), DPPH (HAT), and ABTS (mixed SET/HAT) [51]. The quantitatively higher scavenging values consistently observed for ABTS compared to DPPH are a result of the ABTS radical’s ability to capture radicals through both SET and HAT pathways.

Fish reared at low temperatures exhibited lower radical-scavenging activity, whereas those exposed to high temperatures at any point during growth showed elevated antioxidant activity. The lower antioxidant activity at low temperature indicates a healthy metabolic state [19]. As this study mimics seasonal shifts through temperature and photoperiods, the suppressed antioxidant activity at low temperatures mirrors physiological adaptation of salmon in cold environments. At lower temperatures, reduced metabolic demand generates minimal ROS; therefore, the muscle does not need to synthesize or mobilize antioxidant defences to protect the fillet. This matches with findings in the published literature where cold-water adaptation lowers active antioxidant compounds in the muscle [18,19].

On the other hand, higher temperature can increase ROS formation, which subsequently can increase oxidative stress [6,19,52]. Higher oxidative stress at high temperatures is a direct biochemical consequence of the fish’s accelerated metabolic rate which increases mitochondrial respiration [53]. During this hyper-metabolic state, increased electron flux through the mitochondrial electron transport chain inevitably leads to a higher rate of electron leakage. These escaped electrons prematurely react with oxygen to form ROS [54,55]. Consequently, these ROS cause oxidative stress and when stress exceeds the muscle antioxidant defence capacity, it was shown to increase lipid peroxidation in juvenile seabass muscle [55]. Once active metabolic defences cease after harvest, this accumulated oxidative stress accelerates the degradation of these fatty acids during storage, ultimately generating rancid off-flavours [56].

Implementing the H→L thermal shift produced less than 1% increase (25→26%) in antioxidant activity whereas L→H group produced around 10% increase (17→27%) (Table 5). This indicates that any exposure to high temperatures leaves a lasting oxidative footprint in the muscle. Therefore, avoiding high-temperature stress entirely and maintaining low temperatures is necessary to minimize ROS generation, protect PUFAs from future peroxidation, and provide stable, nutritionally sound fillets.

## 4. Conclusions

Fish gained weight faster and accumulated more total lipid content at higher growing temperatures, which is important for producing marketable-sized fish in a shorter time. However, high temperatures reduced PUFA and increased SFA content in fish muscle, which can affect the desirable nutritional quality of salmon fillet. After switching temperatures, the H→L group showed an increase in PUFA content in the polar lipids, whereas the L→H group showed a decrease. High temperature also produced higher antioxidant activity, which can cause oxidative stress and lipid oxidation in the fillet during storage. After switching the temperature, fish reared in H→L condition showed similar or lower antioxidant activity than previous high temperature condition, whereas L→H group showed higher antioxidant activity.

To bridge the gap between production efficiency and fillet nutritional quality, this study provides evidence for the efficacy of changing temperature. While rearing salmon initially at high temperatures increases neutral lipid accumulation, executing a targeted shift from high to low temperatures (H→L) prior to harvest successfully stimulates homeoviscous adaptation, partially recovering the membrane proportions of high-value PUFAs. By adopting these temperature-shift protocols, the aquaculture industry can sustainably deliver a product that is both economically viable to produce and nutritionally sound to consume.

While this study provides valuable insights regarding intensive salmon rearing, several limitations should be noted for future research. First, due to limited space and logistical constraints in the facility at the time of the experiment, rearing treatments were restricted to a single tank for each treatment, which limited the statistical ability to fully isolate true treatment effects from potential tank effects. Second, the experimental design coupled temperature and photoperiod regimes to mimic seasonal effects. Because these variables were not evaluated as independent factors, it is difficult to statistically decouple the isolated effects of temperature from those of extended photoperiod. Third, the study lacked acute stress biomarker profiling, such as plasma cortisol or glucose measurements, which would provide a more comprehensive understanding of the observed oxidative stress. Lastly, random sampling yielded an uneven distribution of male and female fish across the treatment groups. Future studies should ensure a strictly balanced sex ratio to better account for gender-specific physiological responses.

Future studies should investigate the molecular mechanisms driving changes in lipid composition, specifically focusing on the transcriptomic expression of fatty acid desaturase and elongase enzymes during the temperature switching phase. Additionally, it is crucial to evaluate how these thermal interventions affect the long-term sensory profile, shelf life, and post-mortem lipid oxidation kinetics of the fillets during storage.

## Figures and Tables

**Table 1:** Growth performance of salmon reared under four temperature–photoperiod regimes: Low (14 °C, 12 h light, 302 days), High (21 °C, 24 h light, 302 days), Low-to-High (L→H) temperature shift (14 °C, 12 h light→21 °C, 24 h light; 362 days), and High-to-Low (H→L) temperature shift (21 °C, 24 h light→14 °C, 12 h light; 362 days). Mean body weight, total length, condition factor (CF), feed conversion ratio (FCR), specific growth rate (SGR), and gonadosomatic index (GSI) are presented as mean ± SD (*n* = 3). Different letters (a, b, c) indicate statistically significant differences among treatments (*p* < 0.05).

Temperatures	Low	High	Low-to-High	High-to-Low
Mean weight (g)	184.20 ± 9.63 b	328.43 ± 23.91 a	340.36 ± 23.61 a	420.96 ± 58.73 a
Length (cm)	27.33 ± 1.04 c	30.9 ± 0.17 b	30.90 ± 0.84 b	33.73 ± 0.75 a
SGR (%)	1.20 ± 0.3 ab	1.75 ± 0.19 a	1.28 ± 0.43 a	0.41 ± 0.35 b
FCR	0.94	0.82	0.70	1.14
CF (%)	0.90 ± 0.06 b	1.11 ± 0.06 a	1.15 ± 0.01 a	1.09 ± 0.08 a
Male GSI (%)	0.03	0.25 ± 0.11	nd	nd
Female GSI (%)	0.23 ± 0.05	0.34	0.37 ± 0.05 a	0.22 ± 0.02 b

nd = not detected. Statistical analysis was not performed for FCR (calculated at the tank level) and GSI (insufficient biological replicates per sex resulting from random sampling). Therefore, no superscript letters are assigned to these parameters.

**Table 2:** Muscle lipid content and fatty acid composition of salmon reared under four temperature regimes: Low (14 °C, 12 h light, 302 days), High (21 °C, 24 h light, 302 days), Low-to-High (L→H) temperature shift (14 °C, 12 h light→21 °C, 24 h light; 362 days), and High-to-Low (H→L) temperature shift (21 °C, 24 h light→14 °C, 12 h light; 362 days). SFA: Saturated Fatty Acids, MUFA: Monounsaturated Fatty Acids, PUFA: Polyunsaturated Fatty Acids. Values are presented as mean ± SD (*n* = 3). Different letters (a, b, c) indicate statistically significant differences among treatments (*p* < 0.05).

	Temperatures			
Variable	Low	High	Low-to-High	High-to-Low
**Lipid content (%)**				
Total lipids	2.73 ± 0.83 b	4.65 ± 0.49 ab	5.26 ± 1.23 ab	6.89 ± 1.49 a
Neutral lipids	1.66 ± 0.62 b	3.56 ± 0.45 ab	4.05 ± 0.83 a	5.68 ± 1.29 a
Polar lipids	0.85 ± 0.10 a	0.85 ± 0.07 a	0.87 ± 0.11 a	0.90 ± 0.13 a
**Neutral lipids**				
Fatty acids content (%)				
C14:0	4.07 ± 0.09 a	4.05 ± 0.04 a	3.5 ± 0.09 b	3.64 ± 0.12 b
C16:0	20.30 ± 0.49 a	20.55 ± 0.71 a	19.83 ± 0.61 a	19.67 ± 0.46 a
C16:1n7	5.85 ± 0.12 a	5.82 ± 0.18 a	5.19 ± 0.12 b	5.46 ± 0.10 b
C18:0	5.46 ± 0.22 a	5.69 ± 0.16 a	5.67 ± 0.04 a	5.90 ± 0.25 a
C18:1n9	31.90 ± 0.69 a	31.59 ± 0.14 a	31.83 ± 0.30 a	31.99 ± 1.49 a
C18:1n7	3.18 ± 0.04 a	3.04 ± 0.07 a	3.06 ± 0.10 a	3.10 ± 0.06 a
C18:2n6	11.26 ± 0.43 b	11.40 ± 0.43 b	13.37 ± 0.39 a	12.2 ± 0.20 b
C18:3n3	0.96 ± 0.03 b	0.98 ± 0.03 b	1.10 ± 0.03 a	1.03 ± 0.01 ab
C18:4n3	0.79 ± 0.01 ab	0.84 ± 0.02 a	0.74 ± 0.02 b	0.78 ± 0.06 ab
C20:0	0.22 ± 0.01 a	0.20 ± 0.00 a	0.22 ± 0.01 a	0.24 ± 0.03 a
C20:1n9	2.01 ± 0.06 a	1.91 ± 0.00 a	1.97 ± 0.16 a	2.03 ± 0.12 a
C20:2n6	0.97 ± 0.06 a	0.84 ± 0.06 b	0.91 ± 0.04 ab	0.91 ± 0.03 ab
C20:3n6	0.65 ± 0.01 a	0.65 ± 0.02 a	0.65 ± 0.01 a	0.66 ± 0.03 a
C20:4n6	0.82 ± 0.06 a	0.75 ± 0.02 a	0.73 ± 0.01 a	0.72 ± 0.05 a
C20:4n3	0.48 ± 0.04 a	0.47 ± 0.04 a	0.41 ± 0.02 a	0.45 ± 0.04 a
C20:5n3	2.76 ± 0.13 a	2.81 ± 0.11 a	2.58 ± 0.06 a	2.71 ± 0.23 a
C22:4n6	0.12 ± 0.02 b	0.16 ± 0.00 a	0.14 ± 0.01 ab	0.15 ± 0.01 a
C22:5n6	0.28 ± 0.04 a	0.30 ± 0.00 a	0.26 ± 0.01 a	0.29 ± 0.01 a
C22:5n3	1.16 ± 0.05 a	1.27 ± 0.07 a	1.16 ± 0.03 a	1.23 ± 0.03 a
C22:6n3	6.56 ± 0.31 a	6.48 ± 0.10 a	6.36 ± 0.17 a	6.62 ± 0.36 a
C24:1n9	0.20 ± 0.04 b	0.20 ± 0.00 b	0.32 ± 0.02 a	0.23 ± 0.05 b
Fatty acids class				
∑ SFA	30.05 ± 0.64 a	30.50 ± 0.79 a	29.22 ± 0.66 a	29.46 ± 0.60 a
∑ MUFA	43.14 ± 0.89 a	42.56 ± 0.22 a	42.37 ± 0.64 a	42.80 ± 1.46 a
∑ PUFA	25.06 ± 0.28 b	25.12 ± 0.53 b	26.57 ± 0.26 a	25.93 ± 0.82 ab
∑ n-3	12.71 ± 0.21 a	12.85 ± 0.25 a	12.35 ± 0.24 a	12.82 ± 0.62 a
∑ n-6	14.10 ± 0.38 b	14.10 ± 0.48 b	16.06 ± 0.37 a	14.93 ± 0.25 b
n-3/n-6	0.90 ± 0.04 a	0.91 ± 0.03 a	0.77 ± 0.03 b	0.86 ± 0.03 a
PUFA/SFA	0.83 ± 0.01 b	0.82 ± 0.04 b	0.91 ± 0.02 a	0.88 ± 0.01 ab
**Polar lipids**				
Fatty acids content (%)				
C14:0	1.18 ± 0.00 a	1.32 ± 0.20 a	1.39 ± 0.23 a	1.18 ± 0.07 a
C16:0	21.12 ± 0.68 b	24.21 ± 0.29 a	24.27 ± 0.29 a	24.04 ± 0.48 a
C16:1n7	1.50 ± 0.17 a	1.42 ± 0.22 a	1.54 ± 0.40 a	1.40 ± 0.13 a
C18:0	5.86 ± 0.06 c	6.27 ± 0.12 b	7.35 ± 0.15 a	6.30 ± 0.21 b
C18:1n9	10.27 ± 1.17 ab	9.40 ± 1.02 b	12.60 ± 0.97 a	10.85 ± 0.39 ab
C18:1n7	1.81 ± 0.13 a	1.63 ± 0.08 ab	1.76 ± 0.25 ab	1.39 ± 0.08 b
C18:2n6	3.65 ± 0.68 b	3.26 ± 0.40 b	5.03 ± 0.65 a	4.29 ± 0.19 ab
C18:3n3	0.46 ± 0.07 a	0.42 ± 0.04 a	0.50 ± 0.02 a	0.46 ± 0.02 a
C20:0	0.10 ± 0.01 b	0.10 ± 0.01 b	0.34 ± 0.05 a	0.26 ± 0.08 a
C20:1n9	0.34 ± 0.04 a	0.32 ± 0.06 a	0.40 ± 0.06 a	0.38 ± 0.03 a
C20:2n6	0.45 ± 0.06 a	0.44 ± 0.07 a	0.56 ± 0.07 a	0.54 ± 0.17 a
C20:3n6	0.73 ± 0.04 b	0.83 ± 0.13 ab	0.98 ± 0.04 a	0.88 ± 0.11 ab
C20:4n6	2.73 ± 0.09 b	3.02 ± 0.06 ab	3.15 ± 0.33 ab	3.38 ± 0.28 a
C20:4n3	0.49 ± 0.05 a	0.40 ± 0.06 ab	0.29 ± 0.02 b	0.38 ± 0.03 ab
C20:5n3	8.33 ± 0.93 a	8.06 ± 0.18 ab	6.94 ± 0.40 b	7.34 ± 0.12 ab
C22:5n6	0.97 ± 0.04 ab	1.12 ± 0.12 a	0.94 ± 0.04 b	1.09 ± 0.06 ab
C22:5n3	2.03 ± 0.28 a	1.89 ± 0.08 a	1.73 ± 0.24 a	1.79 ± 0.12 a
C22:6n3	37.93 ± 2.79 a	35.87 ± 1.91 a	30.23 ± 1.34 b	34.06 ± 0.49 ab
Fatty acids class				
∑ SFA	28.26 ± 0.74 c	31.91 ± 0.13 b	33.35 ± 0.20 a	31.78 ± 0.58 b
∑ MUFA	13.97 ± 1.57 ab	12.77 ± 1.31 b	16.30 ± 1.66 a	14.02 ± 0.57 ab
∑ PUFA	57.31 ± 1.16 a	54.90 ± 1.26 ab	49.85 ± 1.49 c	53.74 ± 0.64 b
∑ n-3	49.24 ± 1.66 a	46.65 ± 1.76 ab	39.69 ± 1.81 c	44.02 ± 0.64 b
∑ n-6	8.53 ± 0.70 b	8.67 ± 0.76 b	10.66 ± 0.36 a	10.18 ± 0.32 a
n-3/n-6	5.81 ± 0.66 a	5.41 ± 0.66 ab	3.73 ± 0.30 c	4.33 ± 0.16 bc
PUFA/SFA	2.03 ± 0.05 a	1.72 ± 0.03 b	1.49 ± 0.04 c	1.69 ± 0.05 b

**Table 3:** Spatial (head, middle, and tail) variation in lipid content and fatty acid profiles. Values are presented as mean ± SD (*n* = 3). Different letters (a, b) indicate statistically significant differences among different regions in the fillet (*p* < 0.05).

Variable	Head	Middle	Tail
**Lipid content (%)**			
Total lipid	6.08 ± 1.51 a	5.06 ± 0.51 a	2.77 ± 0.77 b
Neutral lipid	4.51 ± 1.38 a	3.80 ± 0.50 a	2.00 ± 0.65 b
Polar lipid	0.90 ± 0.11 a	0.75 ± 0.05 ab	0.73 ± 0.18 b
**Neutral lipid**			
Fatty acid content (%)			
C14:0	3.57 ± 0.12 a	3.59 ± 0.11 a	3.56 ± 0.14 a
C16:0	19.75 ± 0.49 a	19.84 ± 0.39 a	19.91 ± 0.65 a
C16:1n7	5.32 ± 0.18 a	5.31 ± 0.18 a	5.31 ± 0.25 a
C18:0	5.79 ± 0.20 a	5.69 ± 0.28 a	5.89 ± 0.59 a
C18:1n9	31.91 ± 0.97 a	31.9 ± 0.43 a	31.21 ± 0.41 a
C18:1n7	3.08 ± 0.08 a	3.07 ± 0.05 a	3.08 ± 0.09 a
C18:2n6	12.78 ± 0.7 a	12.86 ± 0.63 a	12.84 ± 0.43 a
C18:3n3	1.06 ± 0.04 a	1.06 ± 0.04 a	1.07 ± 0.04 a
C18:4n3	0.76 ± 0.04 a	0.76 ± 0.05 a	0.73 ± 0.07 a
C20:0	0.23 ± 0.02 a	0.23 ± 0.02 a	0.26 ± 0.07 a
C20:1n9	2.00 ± 0.13 a	1.97 ± 0.12 a	1.92 ± 0.13 a
C20:2n6	0.91 ± 0.03 a	0.91 ± 0.05 a	0.86 ± 0.11 a
C20:3n6	0.65 ± 0.02 a	0.65 ± 0.02 a	0.61 ± 0.06 a
C20:4n6	0.73 ± 0.03 a	0.72 ± 0.03 a	0.75 ± 0.03 a
C20:4n3	0.42 ± 0.03 a	0.42 ± 0.04 a	0.41 ± 0.06 a
C20:5n3	2.65 ± 0.16 a	2.61 ± 0.16 a	2.73 ± 0.14 a
C22:4n6	0.14 ± 0.01 a	0.15 ± 0.01 a	0.12 ± 0.03 b
C22:5n6	0.28 ± 0.01 a	0.28 ± 0.03 a	0.28 ± 0.04 a
C22:5n3	1.19 ± 0.04 a	1.18 ± 0.05 a	1.17 ± 0.09 a
C22:6n3	6.49 ± 0.29 b	6.53 ± 0.37 b	7.05 ± 0.30 a
C24:1n9	0.27 ± 0.06 a	0.28 ± 0.05 a	0.22 ± 0.09 a
Fatty acid class			
∑ SFA	29.34 ± 0.58 a	29.35 ± 0.57 a	29.62 ± 1.09 a
∑ MUFA	42.58 ± 1.03 a	42.53 ± 0.62 a	41.75 ± 0.72 a
∑ PUFA	26.32 ± 0.72 a	26.37 ± 0.36 a	26.93 ± 0.51 a
∑ n3	12.58 ± 0.49 ab	12.56 ± 0.55 b	13.16 ± 0.58 a
∑ n6	15.49 ± 0.68 a	15.57 ± 0.57 a	15.46 ± 0.38 a
n3/n6	0.81 ± 0.05 ab	0.81 ± 0.06 b	0.86 ± 0.05 a
PUFA/SFA	0.90 ± 0.03 a	0.90 ± 0.02 a	0.91 ± 0.05 a
**Polar lipid**			
Fatty acid content (%)			
C14:0	1.29 ± 0.19 a	1.36 ± 0.29 a	1.19 ± 0.15 a
C16:0	24.16 ± 0.37 a	24.22 ± 0.88 a	24.05 ± 1.2 a
C16:1n7	1.47 ± 0.28 a	1.50 ± 0.27 a	1.29 ± 0.17 a
C18:0	6.82 ± 0.60 b	6.79 ± 0.52 b	7.24 ± 0.97 a
C18:1n9	11.73 ± 1.16 a	11.83 ± 1.65 a	11.15 ± 1.61 a
C18:1n7	1.57 ± 0.26 a	1.55 ± 0.12 a	1.58 ± 0.23 a
C18:2n6	4.66 ± 0.59 a	4.70 ± 0.70 a	4.12 ± 0.21 b
C18:3n3	0.48 ± 0.03 a	0.51 ± 0.05 a	0.47 ± 0.04 a
C20:0	0.30 ± 0.07 a	0.29 ± 0.08 a	0.38 ± 0.21 a
C20:1n9	0.39 ± 0.04 a	0.43 ± 0.09 a	0.39 ± 0.09 a
C20:2n6	0.55 ± 0.11 a	0.50 ± 0.11 a	0.48 ± 0.05 a
C20:3n6	0.93 ± 0.09 a	0.89 ± 0.10 a	0.92 ± 0.08 a
C20:4n6	3.26 ± 0.30 a	3.21 ± 0.34 a	3.29 ± 0.26 a
C20:4n3	0.33 ± 0.05 ab	0.38 ± 0.06 a	0.30 ± 0.06 b
C20:5n3	7.14 ± 0.34 a	7.16 ± 0.47 a	7.15 ± 0.58 a
C22:5n6	1.01 ± 0.10 a	1.02 ± 0.17 a	0.99 ± 0.13 a
C22:5n3	1.76 ± 0.17 a	1.83 ± 0.07 a	1.82 ± 0.11 a
C22:6n3	32.14 ± 2.28 a	31.82 ± 3.5 a	33.19 ± 3.21 a
Fatty acid class			
∑ SFA	32.56 ± 0.94 a	32.67 ± 1.64 a	32.86 ± 2.23 a
∑ MUFA	15.17 ± 1.67 a	15.32 ± 2.10 a	14.41 ± 1.93 a
∑ PUFA	50.18 ± 2.24 a	49.81 ± 3.64 a	50.60 ± 3.87 a
∑ n3	41.85 ± 2.66 a	41.70 ± 3.92 a	42.94 ± 3.87 a
∑ n6	10.42 ± 0.41 a	10.32 ± 0.28 a	9.79 ± 0.26 b
n3/n6	4.03 ± 0.39 b	4.05 ± 0.48 b	4.38 ± 0.34 a
PUFA/SFA	1.54 ± 0.11 a	1.53 ± 0.19 a	1.55 ± 0.23 a

**Table 4:** Lipid content and fatty acid profile comparison based on sex. Values are presented as mean ± SD (*n* = 3). Different letters (a, b) indicate statistically significant differences between sex (*p* < 0.05).

Variable	Female	Male
**Lipid content (%)**		
Total lipid	3.41 ± 0.87 a	3.49 ± 1.63 a
Neutral lipid	2.32 ± 0.72 a	2.89 ± 1.59 a
Polar lipid	0.85 ± 0.10 a	0.85 ± 0.06 a
**Neutral lipids**		
Fatty acids content (%)		
C14:0	4.04 ± 0.07 a	4.08 ± 0.07 a
C16:0	20.40 ± 0.65 a	20.43 ± 0.42 a
C16:1n7	5.86 ± 0.11 a	5.81 ± 0.18 a
C18:0	5.54 ± 0.33 a	5.60 ± 0.03 a
C18:1n9	32.02 ± 0.50 a	31.46 ± 0.29 a
C18:1n7	3.11 ± 0.11 a	3.11 ± 0.08 a
C18:2n6	11.13 ± 0.47 a	11.52 ± 0.22 a
C18:3n3	0.96 ± 0.03 a	0.98 ± 0.02 a
C18:4n3	0.80 ± 0.02 a	0.82 ± 0.04 a
C20:0	0.21 ± 0.00 a	0.21 ± 0.02 a
C20:1n9	2.00 ± 0.08 a	1.92 ± 0.02 b
C20:2n6	0.88 ± 0.08 a	0.91 ± 0.10 a
C20:3n6	0.64 ± 0.01 a	0.65 ± 0.01 a
C20:4n6	0.78 ± 0.04 a	0.78 ± 0.07 a
C20:4n3	0.45 ± 0.02 a	0.49 ± 0.03 a
C20:5n3	2.77 ± 0.14 a	2.80 ± 0.10 a
C22:4n6	0.14 ± 0.02 a	0.14 ± 0.03 a
C22:5n6	0.31 ± 0.03 a	0.28 ± 0.02 a
C22:5n3	1.22 ± 0.04 a	1.20 ± 0.10 a
C22:6n3	6.49 ± 0.31 a	6.55 ± 0.10 a
C24:1n9	0.20 ± 0.04 a	0.19 ± 0.02 a
Fatty acid class		
∑ SFA	30.21 ± 0.91 a	30.33 ± 0.58 a
∑ MUFA	43.19 ± 0.81 a	42.51 ± 0.29 a
∑ PUFA	24.83 ± 0.25 a	25.35 ± 0.32 a
∑ n3	12.71 ± 0.21 a	12.85 ± 0.24 a
∑ n6	13.90 ± 0.47 a	14.30 ± 0.14 a
n3/n6	0.92 ± 0.05 a	0.89 ± 0.14 a
PUFA/SFA	0.82 ± 0.03 a	0.83 ± 0.02 a
**Polar lipids**		
Fatty acids content (%)		
C14:0	1.27 ± 0.17 a	1.21 ± 0.15 a
C16:0	22.22 ± 1.49 a	22.86 ± 2.29 a
C16:1n7	1.51 ± 0.13 a	1.44 ± 0.26 a
C18:0	5.94 ± 0.14 a	6.12 ± 0.32 a
C18:1n9	9.92 ± 0.88 a	9.64 ± 1.44 a
C18:1n7	1.72 ± 0.08 a	1.70 ± 0.20 a
C18:2n6	3.37 ± 0.46 a	3.49 ± 0.70 a
C18:3n3	0.43 ± 0.06 a	0.44 ± 0.07 a
C18:4n3	0.21 ± 0.02 a	0.19 ± 0.01 a
C20:0	0.09 ± 0.01 a	0.10 ± 0.00 a
C20:1n9	0.33 ± 0.04 a	0.31 ± 0.01 a
C20:2n6	0.43 ± 0.03 a	0.45 ± 0.08 a
C20:3n6	0.75 ± 0.08 a	0.79 ± 0.13 a
C20:4n6	2.79 ± 0.16 a	2.92 ± 0.18 a
C20:4n3	0.44 ± 0.05 a	0.44 ± 0.09 a
C20:5n3	7.87 ± 0.23 b	8.42 ± 0.80 a
C22:4n6	0.17 ± 0.04 a	0.18 ± 0.02 a
C22:5n6	1.01 ± 0.07 a	1.07 ± 0.15 a
C22:5n3	1.90 ± 0.16 a	1.98 ± 0.26 a
C22:6n3	37.33 ± 3.32 a	36.08 ± 1.48 a
C24:1n9	0.21 ± 0.02 a	0.09 ± 0.02 a
Fatty acid class		
∑ SFA	29.54 ± 1.81 a	30.30 ± 2.63 a
∑ MUFA	13.71 ± 1.23 a	13.19 ± 1.93 a
∑ PUFA	56.09 ± 2.43 a	55.86 ± 0.94 a
∑ n3	48.21 ± 2.93 a	47.57 ± 1.37 a
∑ n6	8.53 ± 0.56 a	8.92 ± 0.81 a
n3/n6	5.67 ± 0.72 a	5.36 ± 0.63 a
PUFA/SFA	1.90 ± 0.19 a	1.85 ± 0.19 a

**Table 5:** Antioxidant activity of salmon reared under four temperature regimes: Low (14 °C, 12 h light, 302 days), High (21 °C, 24 h light, 302 days), Low-to-High (L→H) temperature shift (14 °C, 12 h light→21 °C, 24 h light, 362 days), and High-to-Low (H→L) temperature shift (21 °C, 24 h light→14 °C, 12 h light, 362 days). ABTS: 2,2′-Azino-bis (3-ethylbenzothiazoline-6-sulfonic acid, DPPH: 2,2-Diphenyl-1-picrylhydrazyl, FRAP: Ferric Reducing Antioxidant Power. Values are presented as mean ± SD (*n* = 3). Different letters (a, b) indicate statistically significant differences among treatments (*p* < 0.05).

Variable	ABTS (RSA%)	DPPH (RSA%)	FRAP (abs)
Low	17.46 ± 2.20 b	8.37 ± 0.13 b	0.18 ± 0.05 b
High	25.23 ± 1.92 a	10.07 ± 0.43 ab	0.24 ± 0.01 a
Low to high (L→H)	27.4 ± 0.58 a	11.16 ± 0.69 a	0.25 ± 0.01 a
High to low (H→L)	26.13 ± 0.26 a	9.61 ± 0.78 ab	0.22 ± 0.01 ab

## Data Availability

The original contributions presented in this study are included in the article. Further inquiries can be directed to the corresponding author.
